# Digital skills in healthcare

**DOI:** 10.3205/zma001356

**Published:** 2020-11-16

**Authors:** Inga Hege, Daniel Tolks, Sebastian Kuhn, Thomas Shiozawa

**Affiliations:** 1 Universität Augsburg, Medizinische Fakultät, Lehrstuhl Medical Education Sciences, Augsburg, Germany; 2 Klinikum der LMU München, Institut für Didaktik und Ausbildungsforschung in der Medizin, München, Germany; 3 Leuphana Universität Lüneburg, Zentrum für angewandte Gesundheitswissenschaften, Lüneburg, Germany; 4 Universität Bielefeld, Medizinische Fakultät OWL, Digitale Medizin, Bielefeld, Germany; 5 Eberhard Karls Universität Tübingen, Institut für Klinische Anatomie und Zellanalytik, Tübingen, Germany

## Editorial

Our society is undergoing digital change - digital technologies are no longer uncharted territory; instead, dealing with them has become daily practice. In particular, the COVID-19 pandemic has accelerated this change in many areas of society in a very short period of time, and this has also far-reaching consequences for the health care professions and medical training.

The healthcare sector is changing through the increasing use of digital applications, telemedicine or artificial intelligence (AI) applications [1] especially in diagnosis and treatment planning. Moreover, modern information and communication technologies enable e-health [2] and individualized medicine. Patients can obtain information on the Internet and access a wealth of personal health data via health apps [3]. At the same time, the challenge here are to ensure the quality of information, support the orientation of patients in this environment, and guarantee data and privacy protection.

The rapid change to a digitalized healthcare and the associated requirements demands extensive skills from health care professionals in the use of digital technologies, both in training and in the clinical workplace [4]. For example, they must be able to evaluate digital treatment approaches, learn new practical skills or reflect on their attitude to digital healthcare. This is emphasized by the President of the German Rectors' Conference, who called for a structured and obligatory didactic further training of university teachers on these topics [5]. 

These challenges will also have to be met by universities and colleges for healthcare professionals in the future. The key questions are:

- Which kind of digital skills will healthcare professionals need in the future?
- What influence will digitization have on the relationship between patients and healthcare professionals?
- How do we have to adapt undergraduate and further education in the healthcare professions to meet the requirements of a digital society?

This themed issue addresses a number of important aspects of education from different perspectives. One focus of the articles was on the curricular implementation of digital literacy in general, but also specific topics such as AI have been covered. 

A survey among medical deaneries in Switzerland showed that the importance of teaching digital literacy has been recognized and that curricular development is taking place in many places despite some challenges [6].

Curricula have also been developed or are being developed in Germany. One example is the introduction of a longitudinal interdisciplinary elective course "Digital Health" for medical students in year 3 or above at the University of Hamburg [7]. Since 2017, an elective curriculum for the promotion of digital skills has also been successfully implemented and evaluated at the University Medical School of the Johannes Gutenberg University of Mainz [8].

The Justus-Liebig-University of Gießen offers a successful multi-disciplinary seminar on AI for students of medicine and natural sciences [9].

The digital competencies of students with regard to the use of the Internet – also in distinction to the recently frequently published topic of Internet addiction – are addressed in a commentary [10].

Schick et al. deal with the promotion of communicative competencies with the help of digital media in order to improve the quality of medical education and patient care in the long term [11].

The HiGHMed consortium together with the GMA's Digitization Committee examine the situation in medical informatics in a workshop report and call for better collaboration of the various initiatives and innovations [12].

Finally, members of the Federal Representation of medical students in Germany (bvmd) describe the students' perspective on digitization in a commentary [13].

With this promising, broad and interdisciplinary illumination of the teaching of digital skills from different perspectives, this issue provides a basis for discussion that can serve health profession schools and colleges as a basis for the challenges of digitization and can be a starting point for the development of a joint digitization strategy. The aim should be to provide structured teaching of digital skills.

In the future, research into the training strategies implemented with regard to their effectiveness in later working life and an even stronger focus on interprofessional aspects of teaching digital skills would be of interest. 

## Competing interests

The authors declare that they have no competing interests. 
